# Real World Sex Differences in Patients Undergoing Ascending Aortic Aneurysm Surgery—A Systematic Review and Meta-Analysis of Reconstructed Time-to-Event Data

**DOI:** 10.3390/jcm14061908

**Published:** 2025-03-12

**Authors:** Mohammed Al-Tawil, Alexander Geragotellis, Ahmad Alroobi, Mohammad Aboabdo, Doa’a Alaila, Wafaa A. Sulaiman, Nour Ghaben, Heba T. Salim, Christine Friedrich, René Rusch, Assad Haneya

**Affiliations:** 1 Department of Cardiac and Thoracic Surgery, Heart Center Trier, Krankenhaus der Barmherzigen Brüder, 54292 Trier, Germany; 2 Faculty of Health Sciences, University of Cape Town, Observatory, Cape Town 7700, South Africa; 3 Faculty of Medicine, Al-Quds University, East Jerusalem 20002, Palestine; 4 Department of Cardiovascular Surgery, University Hospital of Schleswig-Holstein, 24118 Kiel, Germany; christine.friedrich@uksh.de; 5 Department of Vascular Surgery, University Hospital of Schleswig-Holstein, 24118 Kiel, Germany

**Keywords:** ascending aortic surgery, ascending aortic aneurysm, sex, gender

## Abstract

**Background:** Men are known to have a higher incidence of acute cardiovascular events, while women are recognized for their increased mortality following diagnosis or intervention for these conditions. The aim of this study is to explore the sex differences in clinical profiles and outcomes of patients undergoing ascending aortic aneurysm (AscAA) surgery. **Methods:** A PRISMA compliant literature search and data extraction were conducted using PubMed, EMBASE, and SCOPUS. Observational cohort or retrospective registries that compared a defined number of male and female adults undergoing ascending aortic surgery for AscAA were included. Data analysis was conducted in compliance with Cochrane methods. **Results:** A total of 11 unique studies met the inclusion criteria, from which 13636 patients were included, with a distribution of 9124 males (67%) and 4512 females (33%). Overall, 91% underwent elective surgery. Male patients had significantly lower 30-day mortality, (RR: 0.68, 95% Cl [0.57, 0.81], *p* < 0.0001) and shorter stays in the intensive care unit, with a mean difference (MD) of −0.48 days ([−0.84, −0.13], *p* = 0.008). Males were significantly younger at the time of surgery (MD: −3.94 years, 95% CI [−5.58, −2.31], *p* < 0.00001). Male patients had significantly more frequent concomitant CABG (21% vs. 14.5%; *p* < 0.0001), while females had more frequent isolated supra-coronary ascending aortic replacement (22% vs. 36%; *p* = 0.004). Female patients who underwent AscAA surgery had significantly lower long-term survival (HR: 1.25, [1.05, 1.50], *p* = 0.013). **Conclusions:** Women undergo surgery for AscAA at older ages and face greater mortality. The disparities in preoperative age and timing of surgery between males and females can be explained by differences in comorbidity profiles and the need for concomitant surgery.

## 1. Introduction

There is a growing academic and clinical interest in sex differences in cardiovascular disease and cardiothoracic surgery. While men are known to have a higher incidence of acute cardiovascular diseases, women are recognized for their increased mortality risk following diagnosis or intervention for these conditions [1,2,3]. The Society of Thoracic Surgeons (STS) and EuroSCORE II risk prediction models assign higher risk probabilities to female patients undergoing bypass or valve surgery [4,5]. Several previous meta-analyses have investigated sex differences in type A aortic dissection, showing relatively similar mortality rates and major postoperative complications [6,7]. However, there continues to be an evidence gap related to proximal aortic disease, particularly ascending aortic aneurysm, and elective cases. It has been established that the growth rate of thoracic aortic aneurysms is three times faster in women compared to men, a phenomenon theorized to be associated with hormonal changes during menopause [8,9,10]. This accelerated growth rate is concerning, as it directly increases the risk of acute and catastrophic aortic events. Furthermore, women with proximal aortic pathologies exhibit higher rates of life-threatening events and in-hospital mortality compared to their male counterparts [11,12]. The established criterion for surgical intervention in patients with AscAA is an ascending aortic diameter exceeding 55 mm [13,14]. However, current guidelines do not address sex-specific differences in AscAA, including possible variations in progression rates, perioperative evaluation, and post-surgery prognosis [13,14]. In this systematic review and meta-analysis, our primary aim was to investigate the influence of sex on mortality in patients undergoing proximal aortic surgery. It also aims at exploring the sex-specific baseline profiles and operative approaches in those patients.

## 2. Methods

### 2.1. Literature Search

We developed search terms using the Population, Intervention, Comparator, and Outcome (PICO) framework to identify relevant studies reporting sex-stratified comparisons of clinical profiles and outcomes in adults. Our search included multiple databases such as PubMed, EMBASE, SCOPUS, and Cochrane, covering studies up to 2 August 2024. This study followed the guidelines of the Updated 2020 Version of the Preferred Reporting Items for Systematic Reviews and Meta-Analyses (PRISMA) [15]. The search terms included “ascending” or “proximal”, “aortic”, “sex” or “gender”, “male” and “female”, and “men” and “women”. To enhance our search results, we performed thorough backward and forward citation checks to ensure the inclusion of all relevant studies. The protocol of this study was registered to PROSPERO (Registration number: CRD42024597273).

### 2.2. Study Selection

The final selection of studies was based on predetermined inclusion and exclusion criteria. During the abstract and full-text review stages, two independent reviewers meticulously evaluated each study, with any discrepancies resolved by a third reviewer. The inclusion criteria for studies were as follows: (1) the studies must compare the clinical profiles and outcomes in both sexes; (2) the number of study subjects for the male and female groups should be clearly defined; (3) only studies involving human participants should be included; (4) the published studies must be in English; (5) all study participants must be adults (age > 18 years); (6) the study designs must employ observational cohort- or registry-based analysis; and (7) abstracts should be included if they meet all the previously mentioned criteria and are the only available source of information. The exclusion criteria for studies were as follows: (1) studies published in languages other than English; (2) studies that do not provide sex-stratified data for clinical profiles and outcomes; and (3) study designs employing systematic reviews, meta-analyses, narrative reviews, case reports/series, editorials, study protocols, and abstracts that do not meet the inclusion criteria.

### 2.3. Data Extraction and Quality Assessment

Data extraction was performed by two independent investigators. To ensure accuracy and consistency, revisions were conducted, and discrepancies were resolved by two additional reviewers. For categorical data, event and total numbers were extracted for each group, while continuous data were recorded as means and standard deviations (SD). If continuous data were reported in other formats, the method employed by Wan et al. [16] was used to estimate the mean and SD. Key details extracted included the study primary outcomes, such as 30-day mortality rates, neurological complications (stroke/TIA), chest tube bleeding (mL), re-thoracotomy, ascending aortic reoperation, new AKI or new dialysis, new myocardial infarction, invasive ventilation time (h), the length of hospital stay (mean ± SD), and the length of ICU stay (mean ± SD). Supporting outcomes such as post-surgery complications were also considered. Those included neurological complications (stroke/TIA), chest tube bleeding (ml), re-thoracotomy, ascending aortic reoperation, new AKI or new dialysis, new myocardial infarction, the length of hospital stay (mean ± SD), and the length of ICU stay (mean ± SD).

Secondary exploratory analysis was aimed at investigating sex differences in baseline patient presentation as well as operative details. Baseline patient profiles included age (mean ± SD), absolute ascending aortic diameter (mean ± SD) (mm), indexed/normalized ascending aortic diameter (mean ± SD) (mm/m^2^), prevalence of bicuspid aortic valve, and the presence of aortic stenosis, aortic regurgitation/insufficiency, hypertension, or diabetes. Operative data included urgent and elective cases, valve-sparing root replacement, isolated supracoronary aortic replacement, concomitant CABG, concomitant AVR, total arch replacement, the operation time (mean ± SD), circulatory arrest/cardioplegia (mean ± SD), the CPB bypass time (mean ± SD), and the aortic cross-clamp time (mean ± SD).

For long-term survival analysis, we used the functions from the R package “IPD from KM”. First, raw data points, including time and survival probabilities, were extracted from Kaplan–Meier curves for each arm (male and female) using the software DigitizeIt Version 2.5.9. In the second step, these data points, along with the numbers at risk, were used to reconstruct individual patient data (IPD) for each arm. The reconstructed datasets from all included studies were then combined into a single dataset, enabling recalculation of aggregated survival curves and risk tables to simulate a patient-level meta-analysis.

The quality of the included studies was assessed using the Newcastle–Ottawa Scale. An adequate follow-up period was defined as at least one year, with a loss to follow-up of no more than 20% within that time frame.

### 2.4. Statistical Analysis

This meta-analysis followed the guidelines of the Cochrane Collaboration and the Meta-Analysis of Observational Studies in Epidemiology (MOOSE) [17]. Data analysis was carried out using Review Manager (Cochrane) and R software (meta and metafor packages). The Mantel–Haenszel method and the random effects model were used to calculate the risk ratio (RR) with 95% confidence intervals (CI) for binary outcome measures. To assess statistical heterogeneity, we used the Q-test for heterogeneity (Cochrane, 1954) and I^2^ statistics. An I^2^ value above 50% indicated high heterogeneity among the included studies. Statistical significance was defined as a *p*-value less than 0.05. To test the robustness of our results, a sensitivity analysis was performed for our primary outcome. A subgroup analysis was planned based on the population characteristics of each study. Studies that included only AscAA were classified as the first group. Studies that included patients with AscAA alongside other conditions, such as chronic dissections or intramural hematomas, were analyzed in a separate subgroup. A secondary exploratory analysis was conducted on preoperative and operative details to understand potential differences in baseline profiles and the surgical approaches indicated on the ascending aorta.

## 3. Results

### Included Studies

The literature search yielded 725 articles. After 283 duplicates were removed, 441 unique articles underwent title and abstract screening. Eventually, 11 unique studies met our inclusion criteria [11,12,18,19,20,21,22,23,24,25,26]. Six studies included exclusively AscAA patients, while the other five included patients who underwent proximal aortic surgery, with a majority concerning ascending and thoracic aneurysms as well as a mix of chronic dissections and intramural hematomas. A PRISMA flowchart highlighting the study selection is available in the Supplementary Material (Figure S1). A total of 13,636 patients were included, and 9124 males (67%) and 4512 females (33%) were contrasted. Table 1 shows a brief description of the included studies and the reported survival rates. Risk of bias assessment results are summarized in Table S3. Overall, nine studies were classified as being of high quality, while two studies were rated as being of moderate quality.

## 4. Primary Outcome Analysis

**Figure 1:**
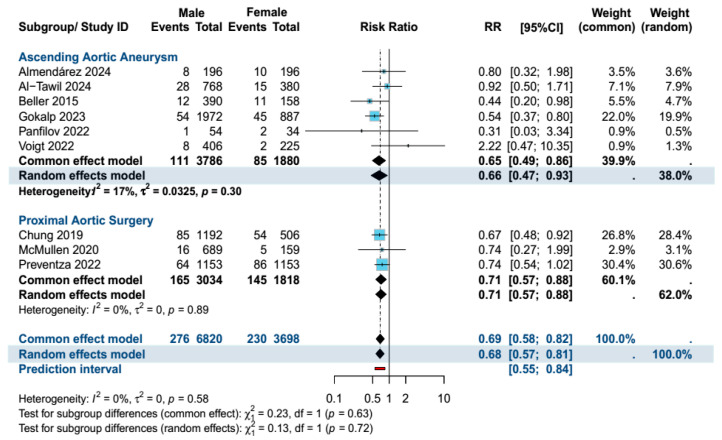
Forest plot of the primary outcome (30-day mortality) summarizing the results of the analysis [11,12,18,19,20,21,22,23,24,25,26].

**Figure 2:**
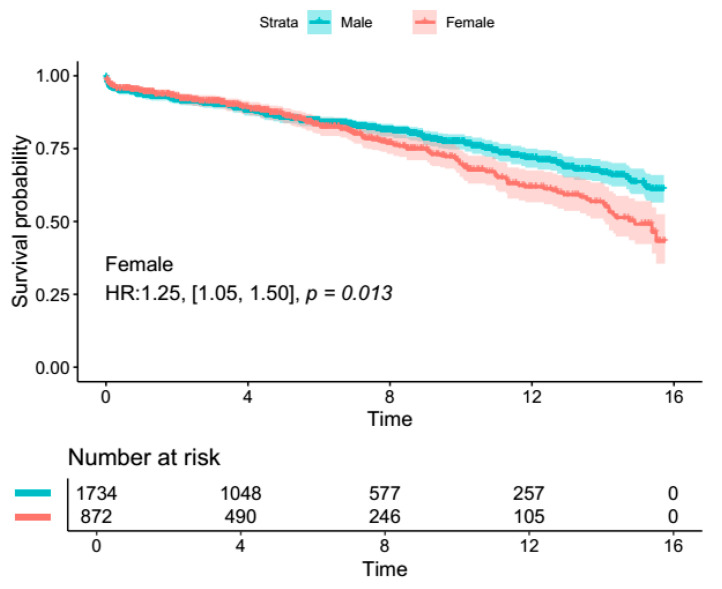
Survival analysis from reconstructed time-to-event data from four included studies [18,19,20,22].

**Figure 3:**
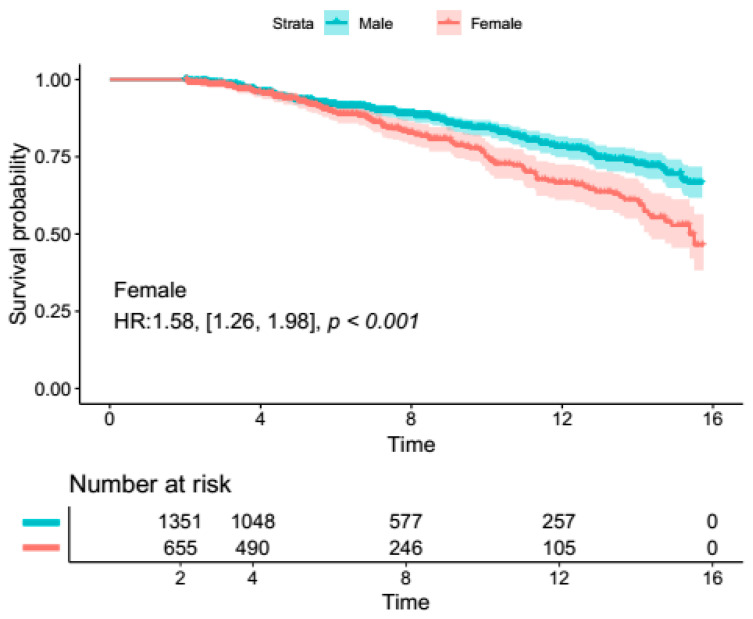
Two-year landmark survival analysis from reconstructed time-to-event data from four included studies [18,19,20,22].

Male patients had significantly lower 30-day mortality in the overall population (RR: 0.68, 95% Cl [0.57, 0.81], *p* < 0.0001), as well as in the AscAA subgroup (RR: 0.66, 95% CI [0.47, 0.93], *p* = 0.02). There were no differences in terms of neurological complications (stroke/TIA) and reoperation rates (Figure 1). The results remained resistant to change upon sensitivity analysis. Table S2 summarizes the findings of the sensitivity analysis for mortality. The length of hospital stay was significantly longer in female patients (MD: 0.58, 95% Cl [0.12, 1.03], *p* = 0.01). Female patients also had significantly longer ICU stays (MD: 0.48, 95% Cl [0.13, 0.84], *p* = 0.008). However, no difference in ICU or hospital length of stay was observed in the AscAA subgroup. In terms of long-term survival, reconstructed time-to-event data of patients who underwent AscAA showed significantly lower long-term survival in female patients (15-year survival: 63.6% vs. 49.1%, *p* < 0.001) (Figure 2 and Figure 3).

### 4.1. Secondary Outcomes (Overall Proximal Aortic Surgery)

Male patients were significantly younger at the time of surgery (mean difference (MD): −3.94, 95% CI [−5.58, −2.31], *p* < 0.00001). Absolute AscAA diameter did not exhibit significant differences between males and females. When indexed to body surface area, the normalized AscAA diameter was significantly larger in female patients at the time of surgery (MD: 3.19 95% CI [2.31, 4.06], *p* < 0.00001). Table 2 provides a comprehensive summary of our meta-analysis results.

In terms of cardiovascular co-morbidities, male patients had significantly more frequent bicuspid aortic valve (RR: 1.51, 95% CI [1.09, 2.08], *p* = 0.01). There were no significant differences in terms of aortic stenosis, aortic insufficiency, and diabetes mellitus between sexes. Female patients presented with higher rates of hypertension (RR: 0.94, 95% CI [0.88, 1.00], *p* = 0.05).

In terms of operative details, there were no significant differences between sexes in terms of urgent and elective cases, valve-sparing root replacement, and concomitant aortic valve replacement. Male patients underwent less frequent total arch replacement when compared to females (RR: 0.67, 95% CI [0.50, 0.91], *p* = 0.009). However, male patients had significantly higher rates of concomitant CABG (RR: 1.46, 95% CI [1.21, 1.77], *p* < 0.001).

Regarding operative time, male patients had significantly longer operative times (MD: 16.60 min, 95% CI [1.32, 31.87], *p* = 0.03), aortic cross-clamp times, (MD: 10.36 min, 95% CI [4.50, 16.21], *p* < 0.001), and pump times (MD: 10.61 min, 95% CI [4.55, 16.68], *p* < 0.001). In terms of post-surgery outcomes, male patients had significantly lower 30-day mortality (RR: 0.68, 95% Cl [0.57, 0.81], *p* < 0.001). There were no differences in terms of stroke/TIA, reoperation, new acute kidney injury, new myocardial infarction, or ventilation time. Male patients had more frequent re-thoracotomy when compared to females (RR: 1.11, 95% Cl [1.00, 1.23], *p* = 0.05).

### 4.2. Secondary Outcomes (Ascending Aortic Aneurysm Surgery Subgroup)

In the AscAA subgroup, male patients were significantly younger (mean difference: −4.25 years, 95% CI [−6.94, −1.55], *p* < 0.001). Female patients had larger absolute AscAA diameters at the time of surgery (MD: −0.74, 95% CI [−1.73, 0.24], *p* = 0.14), but results did not reach significance. When indexed to body surface area, the normalized AscAA diameter was significantly larger in female patients at the time of surgery (MD: 3.21, 95% CI [1.84, 4.58], *p* < 0.001).

In terms of cardiovascular co-morbidities, male patients had more frequent bicuspid aortic valve (RR: 1.36, 95% CI [1.00, 1.85], *p* = 0.05). There were no significant differences in terms of other valvular and cardiovascular comorbidities. Figure 2 and Figure 3 summarize the highlights of sex differences in the patients who underwent surgery for AscAA.

No significant differences were noted in terms of indication for urgent surgery, valve-sparing root replacement, or concomitant aortic valve replacement. Male patients underwent less frequent isolated supracoronary aortic replacement (RR: 0.67, 95% CI [0.51, 0.88], *p* = 0.004) and total arch replacement (RR: 0.49, 95% CI [0.25, 0.98], *p* = 0.04). However, they had significantly higher rates of concomitant CABG (RR: 1.64, 95% CI [1.19, 2.26], *p* = 0.003). Table 2 provides a comprehensive summary of the analysis results. The absolute proportions are also reported to give context to the results.

## 5. Discussion

Sex-related differences in cardiovascular diseases are increasingly gaining attention. Suboptimal results have been described post-coronary surgery and valve surgery in females, as well as for abdominal aortic aneurysm [27,28]. Our meta-analysis sought to identify sex-based differences in outcomes among patients undergoing proximal aortic surgery. Current guidelines indicate ascending aortic replacement based on the diameter of the aorta, due to the well-documented impact of aortic size on aortic-related outcomes and death. Irrespective of sex, the “borderline” for non-syndromic patients is ≥55 mm [13,29] based on Elefteriades’ [30] description of a “hinge point” at 60 mm, when the risk of rupture or dissection increases dramatically.

Our meta-analysis found that female patients had significantly greater 30-day mortality than males. Current estimates of male–female early post-operative mortality differences in ascending aortic surgery are conflicting [11,12,23,25,31], with many studies conducted in single centers with limited sample sizes and clinically divergent patient populations, such as in emergency surgery [12,21,23,25]. To contextualize the results of our analysis, it must be noted that for both the overall and AscAA groups, female patients were significantly older with significantly larger indexed/normalized AscAA diameters than men. Elsewhere, others have also clinically correlated female sex to either faster aneurysmal growth rates or an independent risk factor for aortic expansion [8,32]. The rapid growth rate of ascending aortic aneurysm tissue in female patients has previously been attributed to a combination of higher activity of matrix metalloproteinases 2 and 9 and lower tissue inhibitor metalloproteinases [33], with further degeneration accelerated by the decrease in endogenous estrogen levels in women of peri- and post-menopausal age [34,35]. There remains scope for further translational studies to investigate pathophysiological differences between male and female patients to guide clinical interventions and decision making based on comorbidities, aneurysm location, and risk factors.

Additionally, our analysis showed longer stays in hospital and intensive care for females. Other adverse in-hospital outcomes were mostly comparable. On a functional level, being aware of these differences is critical, as cautious and preventative measures can be taken throughout the pre-, intra-, and post-operative period. These include emphasis on anesthetic preparation, sterility, surgical technique, and routine post-operative care, such as early catheter removal and mobilization with physiotherapy.

An important limitation of our study is that long-term mortality data were not extrapolated. The current literature is conflicted on the matter, but many recent multivariable analyses do not recognize sex as an independent risk factor for long-term mortality [18,20,22]. For instance, a very recent study by Almendárez et al. [22] found an HR of 0.68 (95% CI 0.43–1.07, *p* = 0.23), using a mean follow-up of 52 ± 35 months. While Voigt et al. [18] found no significant differences, they encouraged larger sample sizes, as their Kaplan–Meier survival analysis confidence intervals were inflated. This was largely due to a low overall number of events/low sample size (630) and violation of the proportional hazard assumption that hindered their ability to quantify the effect of age. Previously, our group studied 1148 patients and found male vs. female survival rates of 88% vs. 88% at 5 years, 76% vs. 71% at 10 years, and 59% vs. 47% at 15 years [20]. Other studies that only performed univariate analyses, thereby retaining confounders, appear to rank females with higher long-term mortality [11].

Undertaking pre-emptive ascending aortic repair has been suggested by Panfilov et al. [19] based on the rapid growth rate of aneurysms and poorer outcomes in female patients. They speculate that the fragility incurred by a larger aortic diameter may hamper aortic reconstruction and may worsen surgical outcomes in women. The realistic introduction of this clinical paradigm relies on risk profiling certain women where the risks of undergoing pre-emptive surgery fall short of the risks of a conservative approach. To this end, efforts to implement robust screening of women reaching perimenopausal age, especially those with documented vascular risk factors, are warranted to identify those patients who may qualify for pre-emptive intervention. There remains a need to formulate sex-specific strategies for AscAA surveillance and treatment.

While specific screening protocols for AscAA are not routinely implemented unless complications arise, aneurysms are often detected incidentally through echocardiography, chest X-rays, or computed tomography performed for other indications. However, closer surveillance of female patients may be warranted, given their larger indexed aortic diameters and the fact that they receive less frequent follow-ups due to a lower burden of comorbidities. Current and past guidelines have recommended surgical intervention for AscAA at diameters as low as 4.5 cm when concomitant cardiac surgery is planned. Notably, men may receive more consistent monitoring and earlier surgery due to their higher prevalence of comorbidities and more frequent bicuspid aortic valve. In contrast, women, who more often present with isolated AscAA and fewer comorbidities, may undergo fewer routine follow-ups and relatively delayed surgery. This disparity raises the hypothesis that earlier intervention in men is primarily driven by their comorbidities rather than aneurysm-related risk alone. Furthermore, the higher comorbidity burden in male patients may skew survival comparisons, potentially masking a more pronounced lower survival rate in female patients that warrants further investigation.

The limitations of this study include the inherent observational nature of the included studies and the lack of control for confounding factors. However, we also view this as a strength, as it reflects real-world data and incorporates various influencing factors. Another limitation is that some studies did not exclude urgent or emergent cases, though less than 9% of the patients overall underwent such procedures. Future large-scale, multicenter studies with in-depth analyses are essential to investigate sex-specific predictors and outcome differences. This is a well-known limitation of aggregate study-level meta-analysis. Additionally, future research would benefit from testing the hypothesis that concomitant surgeries during elective AscAA repair may increase mortality, providing valuable insights for clinical practice.

## 6. Conclusions

Our data identifies that females undergoing ascending aortic surgery, as compared to males, have significantly greater 30-day mortality, longer hospital stays, and longer ICU stays, with non-significant differences noted in post-operative systemic complications. The disparities in preoperative age and the timing of surgery between males and females can be potentially explained by differences in comorbidity profiles and the need for concomitant surgery.

## Figures and Tables

**Table 1:** Overview of the included studies in this meta-analysis. * Bold means significant result.

Study ID	Location	Study Duration	Population	Patients		FU (y)	Long Term Survival		
				Male	Female		Male	Female	*p* Value
Beller et al., 2015 [11]	Germany	1994–2011	Ascending aneurysm	390	158	_	_	_	_
Voigt et al., 2022 [18]	Netherlands	1991–2016	Ascending aneurysm	406	224	15-year	72.9%	48.1%	<0.001 *
Panfilov et al., 2022 [19]	Russia	2013–2021	Ascending aneurysm	54	34	3-year	83.5	94.3%	0.43
Al-Tawil et al., 2024 [20]	Germany	2002–2021	Ascending aneurysm	768	380	15-year	59%	51%	0.15
Gokalp et al., 2023 [21]	Netherlands	2013–2017	Ascending aneurysm	1972	887	_	_	_	_
Almendárez et al., 2024 [22]	Spain	2000–2019	Ascending aneurysm	506	232	8-year	76.1%	75.8%	0.23
Chung et al., 2019 [12]	Canada	2002–2017	Proximal aortic surgery	1155	498	_	_	_	_
McMullen et al., 2020 [23]	USA	2005–2018	Proximal aortic surgery	689	159	5-year	93.6%	91.3%	0.43
Kampen et al., 2022 [24]	Germany	2000–2018	Proximal aortic surgery	1331	442	5-year	91.7%	87.6%	0.02 *
Preventza et al., 2022 [25]	USA	1990–2020	Proximal aortic surgery	1153	1153	5-year	67.1%	66.3%	0.40
Vignac et al., 2022 [26]	Sweden	2007–2017	Proximal aortic surgery	700	345	_	_	_	_

**Table 2:** Summary of meta-analysis results.

	Overall Proximal Aortic Surgery				Ascending Aortic Aneurysm Subgroup			
	Patients Analyzed	Effect Estimate	M% vs. F%	*p* Value	Patients Analyzed	Effect Estimate	M% vs. F%	*p* Value
Age (mean ± SD) #	13,637	−3.94 [−5.58, −2.31]	/	*p* < 0.001	6012	−4.25 [−6.94, −1.55]	/	*p* = 0.002
Absolute AscA diameter (mean ± SD) (mm) #	7624	−0.88 [−2.46, 0.69]	/	*p* = 0.27	3153	−0.74 [−1.73, 0.24]	/	*p* = 0.14
Indexed/normalized ascending aortic diameter (mean ± SD) (mm/m^2^) #	7624	−3.19 [−4.06, −2.31]	/	*p* < 0.001	3153	−3.21 [−4.58, −1.84]	/	*p* < 0.001
Bicuspid aortic valve	9014	1.51 [1.09, 2.08]	36% vs. 22%	*p* = 0.01	434	1.36 [1.00, 1.85]	23% vs. 17%	*p* = 0.05
Aortic stenosis	9097	1.16 [0.86, 1.56]	34% vs. 28%	*p* = 0.32	2,517	1.13 [0.84, 1.52]	26% vs. 23%	*p* = 0.41
Aortic regurgitation	9097	1.25 [0.95, 1.64]	43% vs. 30%	*p* = 0.10	2517	0.93 [0.76, 1.15]	43% vs. 48%	*p* = 0.51
Hypertension	12,489	0.94 [0.88, 1.00]	54% vs. 59%	*p* = 0.05	4864	0.90 [0.78, 1.04]	29% vs. 32%	*p* = 0.17
Diabetes	13,637	1.01 [0.88, 1.16]	9% vs. 9%	*p* = 0.85	6012	1.05 [0.87, 1.26]	8% vs. 8%	*p* = 0.63
Operative details								
Elective	7336	1.00 [0.99, 1.01]	90% vs. 91%	*p* = 0.92	4638	1.00 [1.00, 1.00]	98% vs. 98%	*p* = 0.99
Valve-sparing root replacement	9993	1.16 [0.90, 1.49]	11% vs. 10%	*p* = 0.26	5186	1.04 [0.65, 1.67]	8% vs. 8%	*p* = 0.87
Isolated supra-coronary aortic replacement	5924	0.67 [0.51, 0.88]	22% vs. 36%	*p* = 0.004	5924	0.67 [0.51, 0.88]	22% vs. 36%	*p* = 0.004
Concomitant CABG	10,081	1.46 [1.21, 1.77]	21% vs. 14.5%	*p* < 0.001	5274	1.64 [1.19, 2.26]	22% vs. 13%	*p* = 0.003
Concomitant AVR	7960	1.04 [0.85, 1.26]	28% vs. 24%	*p* = 0.72	3153	1.01 [0.84, 1.23]	38% vs. 35%	*p* = 0.88
Total arch replacement	6494	0.67 [0.50, 0.91]	3% vs. 7%	*p* = 0.009	2535	0.49 [0.25, 0.98]	11% vs. 16%	*p* = 0.04
Operation time (mean ± SD) #	3557	16.60 [1.32, 31.87]	/	*p* = 0.03	1784	18.01 [−6.54, 42.56]	/	*p* = 0.15
Circulatory arrest time (mean ± SD) #	7959	−0.73 [−2.74, 1.29]	/	*p* = 0.48	3152	−0.82 [−4.27, 2.62]	/	*p* = 0.64
CPB bypass time (mean ± SD) #	9733	10.61 [4.55, 16.68]	/	*p* < 0.001	3153	9.47 [−1.18, 20.12]	/	*p* = 0.08
Aortic cross-clamp time, (mean ± SD) #	7339	10.36 [4.50, 16.21]	/	*p* < 0.001	3065	9.21 [0.03, 18.39]	/	*p* = 0.05
Postoperative Outcomes								
30-day mortality	10,518	0.68 [0.57, 0.81]	4% vs. 6%	*p* < 0.001	5666	0.66 [0.47, 0.93]	3% vs. 5%	*p* = 0.02
Neurological complications (stroke/TIA)	11,899	0.81 [0.61, 1.07]	5% vs. 4%	*p* = 0.13	5274	0.74 [0.38, 1.43]	4% vs. 6%	*p* = 0.38
Re-thoracotomy	9278	1.11 [1.00, 1.23]	13% vs. 14%	*p* = 0.05	5274	1.03 [0.83, 1.28]	8% vs. 8%	*p* = 0.78
Ascending aortic reoperation	2544	0.99 [0.60, 1.64]	4% vs. 3%	*p* = 0.97	1686	0.76 [0.10, 5.98]	0.5% vs. 0.6%	*p* = 0.79
New AKI, or new dialysis	11,899	0.87 [0.66, 1.15]	5% vs. 6%	*p* = 0.34	5274	0.95 [0.57, 1.58]	5% vs. 5%	*p* = 0.84
New myocardial infarction	8730	1.10 [0.75, 1.60]	1.6% vs. 1.3%	*p* = 0.63	4726	1.34 [0.85, 2.10]	2% vs. 1.6%	*p* = 0.21
New pacemaker	5787	0.92 [0.65, 1.30]	20% vs. 22%	*p* = 0.63	4939	1.04 [0.78, 1.40]	23% vs. 23%	*p* = 0.78
Chest tube bleeding, mL #	1784	0.09 [−0.01, 0.19]	/	*p* = 0.07	1784	0.09 [−0.01, 0.19]	/	*p* = 0.07
Invasive ventilation time, h #	3557	−0.11 [−0.28, 0.06]	/	*p* = 0.22	1784	−0.04 [−0.14, 0.06]	/	*p* = 0.39
Length of hospital stay (mean no. of days) #	11,811	−0.58 [−1.03, −0.12]	/	*p* = 0.01	5186	−0.33 [−1.03, 0.38]	/	*p* = 0.36
Length of ICU stay (mean no. of days) #	9040	−0.48 [−0.84, −0.13]	/	*p* = 0.008	2415	−0.06 [−0.29, 0.18]	/	*p* = 0.64

Binary outcomes reported as the risk ratio and corresponding 95% confidence interval #; continuous outcomes reported as the mean difference and corresponding 95% confidence interval.

## Data Availability

Data derived from public domain resources.

## Supplementary Materials

The following supporting information can be downloaded at: https://www.mdpi.com/article/10.3390/jcm14061908/s1, Table S1: Search strategies across different databases. Figure S1: PRISMA Flowchart. Table S2: Risk of Bias Assessment using the New Castle-Ottawa Scale (Follow-up (FU) length was determined to at least one year, adequacy of FU meant less than 20% loss of patients FU at 12 months). Table S3: Sensitivity Analysis for the primary outcome (30-days mortality). Figure S2: Funnel plot representing studies the studies included in the analysis of the primary outcome (Mortality), Egger’s test showed no significant asymmetry (*p* = 0.52). The study by (Panfilov 2022, bottom left) is the smallest study included, which explains the small study effect caused by it.
