# Supplementary material for: Real World Sex Differences in Patients Undergoing Ascending Aortic Aneurysm Surgery—A Systematic Review and Meta-Analysis of Reconstructed Time-to-Event Data
Source: J Clin Med. 2025 Mar 12;14(6):1908. doi: 10.3390/jcm14061908 (PMC11943001; doi:10.3390/jcm14061908)
Supplement: Supplementary file 1 [file jcm-14-01908-s001.zip › jcm-3470688-supplementary.pdf]

Table S1: Search strategies across different databases.

DataBase	Latest Search Date	Search String	Results by latest search
PubMed	2nd August, 2024	(ascending[title] OR proximal[title]) AND (aortic[title] OR aorta[title]) AND (sex[title/abstract] OR gender[title/abstract] OR (male[title/abstract] AND female[title/abstract]) OR (men[title/abstract] AND women[title/abstract]))	360
Scopus	2nd August, 2024	(TITLE ((ascending OR proximal) AND (aortic OR aorta)) AND TITLE-ABS ((sex OR gender OR (male AND female) OR (men AND women))))	444
Embase	2nd August, 2024	(ascending:ti OR proximal:ti) AND (aortic:ti OR aorta:ti) AND (sex:ti,ab OR gender:ti,ab OR (male:ti,ab AND female:ti,ab) OR (men:ti,ab AND women:ti,ab))	584

Figure S1: PRISMA Flowchart

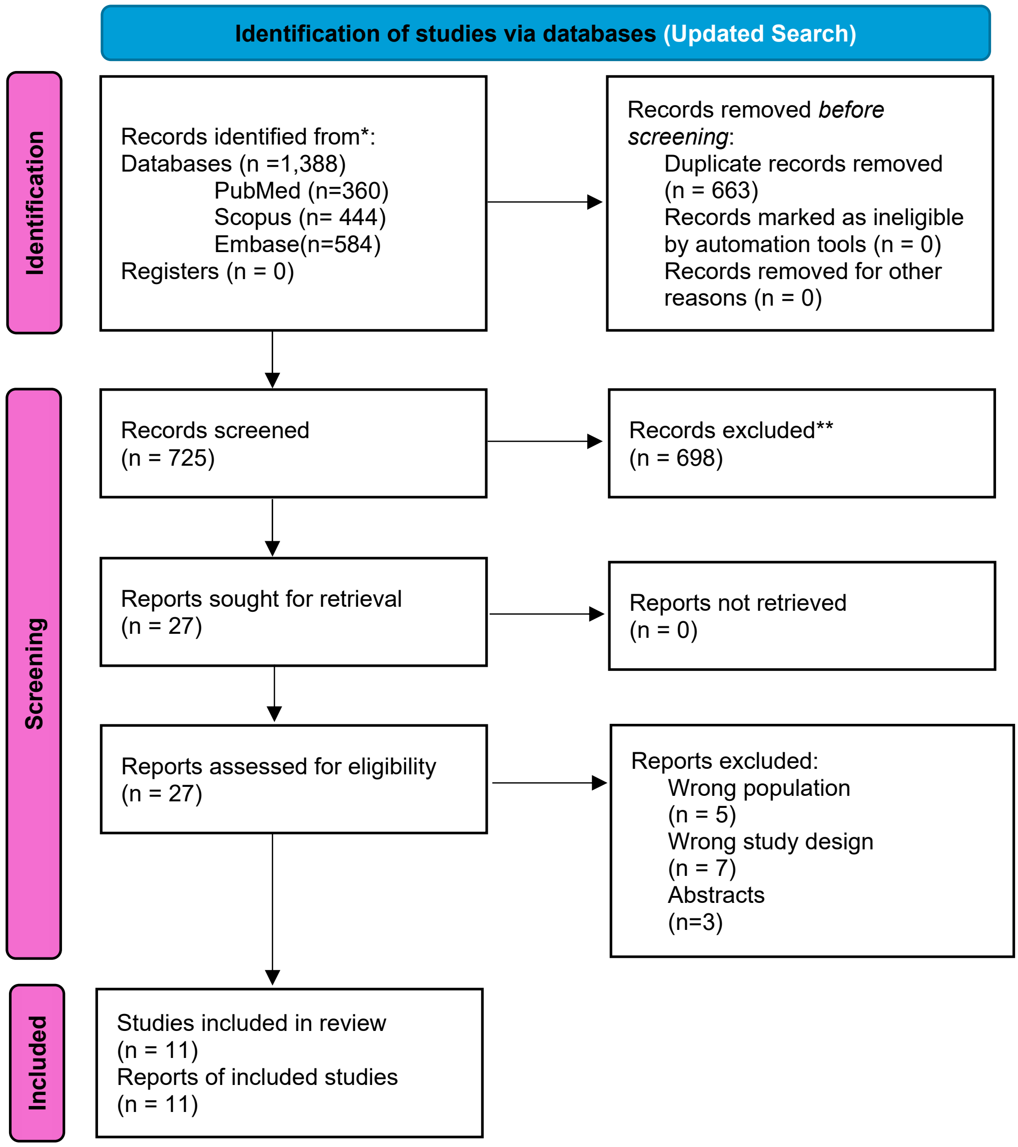

Table S2: Risk of Bias Assessment using the New Castle-Ottawa Scale **(Follow-up (FU) length was determined to at least one year, adequacy of FU meant less than 20% loss of patients FU at 12 months).**

Study ID	Selection	Comparability	Outcome	FU Length	Score	Overall Judgment
Beller et al. 2015 [11]	★★★★	★	★	30-day	6	Moderate
Voigt et al. 2022 [18]	★★★★	★	★★★	15-year	8	Good
Panfilov et al. 2022 [19]	★★★★	★★	★★★	3-year	9	Good
Al-Tawil et al. 2024 [20]	★★★★	★★	★★★	15-year	9	Good
Gokalp et al. 2023 [21]	★★★★	★★	★	30-day	8	Good
Almendárez et al. 2024 [22]	★★★★	★★	★★★	8-year	9	Good
Chung et al. 2019 [12]	★★★★	★★	★	30-day	8	Good
McMullen et al. 2020 [23]	★★★★	★★	★★★	5-year	9	Good
Kampen et al. 2022 [24]	★★★★	★★	★★★	5-year	9	Good
Preventza et al. 2022 [25]	★★★★	★★	★★★	5-year	9	Good
Vignac et al. 2022 [26]	★★★★	★	★	30-days	6	Moderate

Table S3: Sensitivity Analysis for the primary outcome (30-days mortality):

Omitted Study	Total P-value	Subgroup	Influence on Subgroup P value
Almendárez 2024	**p = 0.0000**	**Ascending Aortic Aneurysm**	**p = 0.05**
Al-Tawil 2024	**p = 0.0000**		**p = 0.002**
Beller 2015	**p = 0.0001**		p = 0.10
Gokalp 2023	**p = 0.0009**		p = 0.24
Panfilov 2022	**p = 0.0000**		**p = 0.0007**
Voigt 2022	**p = 0.0000**		**p = 0.04**
McMullen 2020	**p = 0.0000**	**Proximal Aortic Surgery**	**p = 0.003**
Chung 2019	**p = 0.0004**		**p = 0.05**
Preventza 2022	**p = 0.0001**		**p = 0.01**

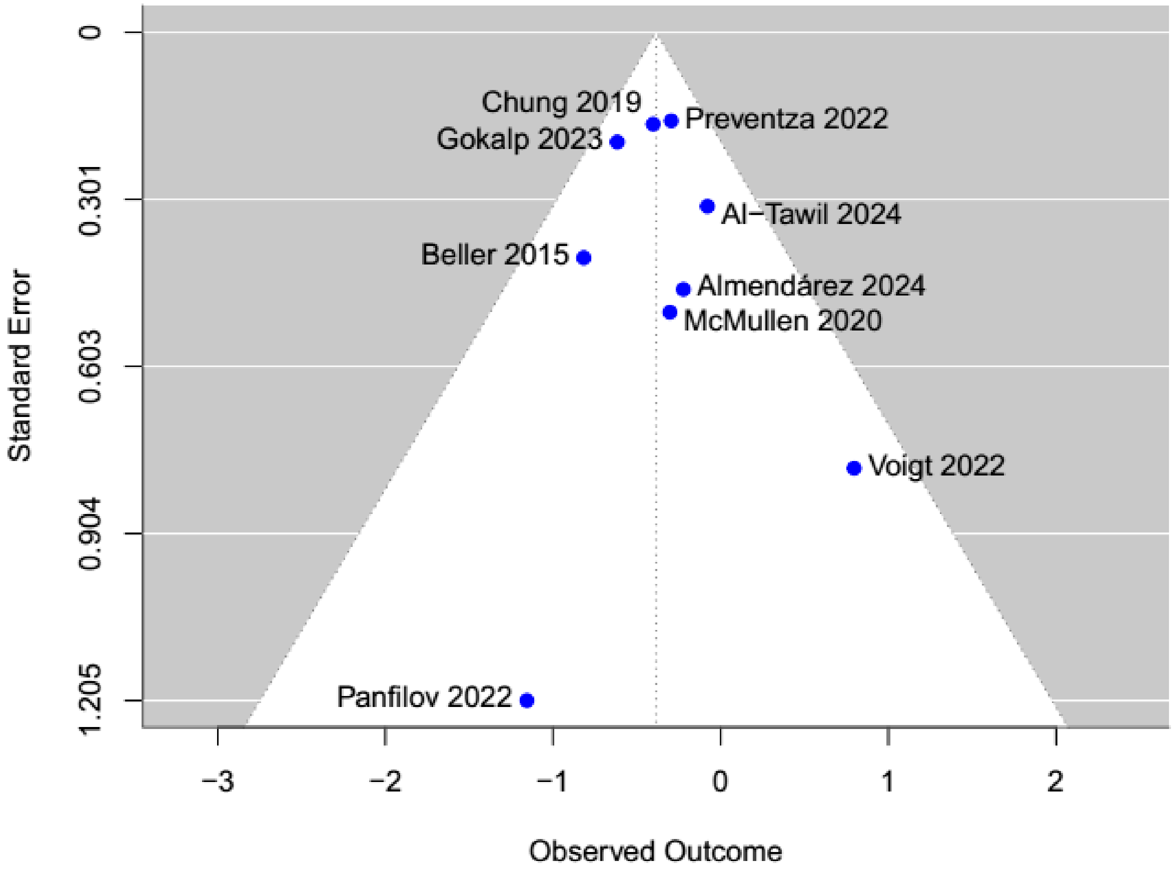

Standard Error
0
0.301
0.603
0.904
1.205
Observed Outcome
−3
−2
−1
0
1
2
Chung 2019
Preventza 2022
Gokalp 2023
Al−Tawil 2024
Beller 2015
Almendárez 2024
McMullen 2020
Voigt 2022
Panfilov 2022

Figure S2: Funnel plot representing studies the studies included in the analysis of the primary outcome (Mortality), Egger's test showed no significant asymmetry (p=0.52). The study by (Panfilov 2022, bottom left) is the smallest study included, which explains the small study effect caused by it.
